# Age‐Dependent Alterations of Chromosomal Passenger Complex Members During Implantation and Decidualization in the Mouse Uterus

**DOI:** 10.1111/acel.70506

**Published:** 2026-04-23

**Authors:** Ezgi Golal, Cemre Nur Balci, Nuray Acar

**Affiliations:** ^1^ Department of Histology and Embryology Faculty of Medicine, Akdeniz University Antalya Turkey; ^2^ Department of Histology and Embryology Faculty of Medicine, Zonguldak Bulent Ecevit University Zonguldak Turkey

**Keywords:** aging, Aurora B, Borealin, implantation, Ki‐67, mouse, pIncenp, Survivin

## Abstract

Successful embryo development, acquisition of uterine receptivity, implantation, and decidualization during the peri‐implantation window are essential events that ensure a healthy pregnancy. While ovarian aging has long been considered the primary cause of age‐related decline in fertility, emerging evidence demonstrates that uterine aging also compromises the ability to support pregnancy. The chromosomal passenger complex, composed of pIncenp, Aurora B, Survivin, and Borealin, is a critical regulator of cell cycle progression, particularly in chromosome condensation, mitotic spindle organization, and cytokinesis. We investigated age‐associated changes in the expression and localization of those members, as well as the proliferation marker Ki‐67, at implantation sites in mice during the peri‐implantation period. Female mice aged 12, 20, and 26–32 weeks were used, and uterine tissues were collected on Days 5, 6, and 8 of pregnancy. Immunohistochemistry was performed to determine the localization of those proteins and Ki‐67, while Western blotting was used to quantify protein expression levels. Our results revealed dynamic and age‐dependent alterations in protein expression throughout pregnancy. Ki‐67 expression decreased with advancing age in the luminal and glandular epithelium on Day 5, whereas pIncenp and Survivin levels were elevated in the stromal compartment of older mice. On Day 6, pIncenp, Borealin, and Survivin expression increased in the luminal epithelium of aging groups, and Aurora B expression was higher in older mice on Day 8. These findings highlight a potential role for complex dysregulation in impaired implantation/decidualization with maternal aging and may provide insight into mechanisms underlying implantation failure and recurrent pregnancy loss.

## Graphical abstract

Aging in females reduces the number of implantation sites and leads to specific, compartment‐dependent changes in components of the chromosomal passenger complex (pIncenp, Aurora B, Survivin, Borealin) during mouse uterine implantation and decidualization. This result suggests that CPC dysregulation may contribute to the age‐related decline in implantation and decidualization.

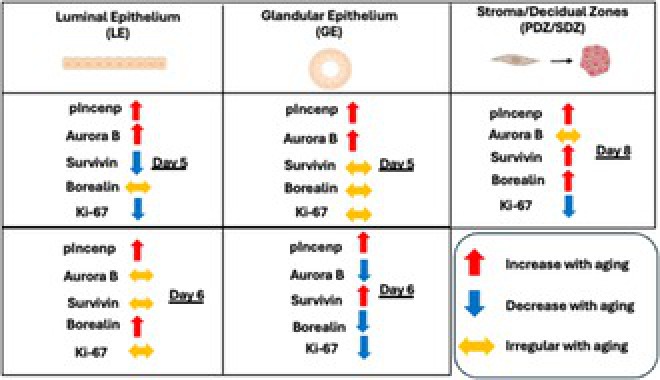

## 1 Introduction

For pregnancy to progress and end healthily, both the healthy development of the blastocyst and the uterus reaching the receptive phase must occur in sync (Li et al. 2015). While oocyte quality is the main factor in fertility, the aging of the uterus also plays a role in reproductive failure (Rosenwaks et al. 1995). Implantation success is lower in older women compared to younger women. Additionally, pregnant women over 45 tend to have higher cesarean section rates. The incidences of gestational diabetes, pre‐eclampsia, placental abruption, placenta previa, postpartum hemorrhage, and preterm birth also rise (Ratiu et al. 2023; Sugai et al. 2023). Aging has been linked to cell cycle arrest, disrupted insulin signaling, dysregulated vascular endothelial growth factor pathways, and inhibited epithelial proliferation (Devesa‐Peiro et al. 2022).

Members of the chromosomal passenger complex (CPC) are proteins that migrate from centromeres to the central part of spindle fibers during mitosis. The chromosomal passenger complex plays a role in chromosome condensation and segregation during mitosis, as well as in completing cytokinesis at the end of mitosis (Adams et al. 2001). The chromosomal passenger complex is a macromolecular assembly that includes the inner centromere protein (INCENP), Aurora B kinase, Survivin (BIRC5), and Borealin (Zhang et al. 2009). INCENP serves as the platform where all members of the complex are assembled (Ainsztein et al. 1998). The N‐terminal of INCENP is necessary for localizing the CPC to centromeres. During mitosis, it relocates from centromeres to spindle fibers and then to the cleavage site (D'Souza et al. 2007). Another component of the complex is Aurora B kinase, which is localized to the centromere and kinetochore (Broad et al. 2020). Aurora B kinase is part of a highly conserved Ser/Thr kinase family (Glover et al. 1995). Another component of the CPC, Survivin (BIRC5), has been suggested to inhibit apoptosis and regulate cell division. Survivin is classified as a member of the inhibitory family of apoptosis proteins (IAPs) due to its N‐terminal baculovirus IAP repeat (BIR) (Ambrosini et al. 1997). However, whether Survivin truly inhibits apoptosis is still debated. Unlike IAPs, Survivin has only a single BIR domain and lacks the RING domain necessary for the anti‐apoptotic function of IAPs (Verhagen et al. 2001). Borealin is a new component of the CPC and has been identified as cell division‐related gene 8 (cdca8, Borealin, Dasra) (Gassmann et al. 2004). The main binding partner of Borealin in the CPC is Survivin, which has structural features of the apoptosis inhibitor protein family (Yang et al. 2004).

Survivin was observed to be localized in villous cytotrophoblasts and syncytiotrophoblasts. Survivin gene expression was found to be decreased in pre‐eclamptic placentas compared to healthy placentas. These findings indicate that Survivin might be essential for maintaining normal placental development and function. Survivin depletion was found to cause arrest in the G2/M phase in trophoblastic cells and inhibit proliferation by inducing apoptosis. Along these lines, research on mitotic regulators has offered additional mechanistic insights. Researchers examined the localization of Aurora B in JAR cells, which are mitotic trophoblast cells, after silencing Survivin. Aurora B stained much weaker at centromeres and kinetochores during metaphase (Muschol‐Steinmetz et al. 2013). This study shows that Survivin and Aurora B may act in concert to regulate chromosomal stability during trophoblast division. In the artificial decidualization model, decidualization was observed to be hindered by the application of the Aurkb inhibitor Barasertib (Wang et al. 2023). These data expand the role of Aurora B beyond mitotic regulation, emphasizing its involvement in reproductive processes such as decidualization. In senescent human dermal fibroblasts and umbilical vessel endothelial cells, Aurora kinase B expression was found to be decreased. The upregulation of Aurora B in these senescent cells partially reversed the aging phenotypes. This suggested that the reduction of Aurora kinase B may contribute to cellular senescence (Kim et al. 2011). Taken together, these findings highlight that the downregulation of Aurora B is not only associated with impaired decidualization but may also play a role in the development of cellular senescence. In the literature, there is no study examining the expressions of the CPC members at the implantation sites during the peri‐implantation period in mice and how these expressions change with aging. In our study, we investigated the localization and expression of CPC members pIncenp, Aurora B, Survivin, and Borealin, as well as the proliferation marker Ki‐67, at the implantation/decidualization sites of 12‐, 20‐, and 26–32‐week‐old mice on Days 5, 6, and 8 of pregnancy.

## 2 Materials and Methods

### 2.1 Animals Groups

All relevant international, national, and institutional regulations concerning the care and use of animals were strictly followed. The experimental protocol was approved by the Animal Care and Use Committee of the Faculty of Medicine at Akdeniz University.

In this study, young, middle‐aged, and old female Balb/C mice obtained from the ‘Experimental Animals Unit’ of Akdeniz University were used. Twelve‐week‐old female mice were included in the young female mice group, 20‐week‐old female mice in the middle‐aged female mice group, and 26–32‐week‐old female mice in the old female mice group (Patel et al. 2017).

Two female mice and one male mouse were housed together overnight for mating, and vaginal plaque was checked the next day. Females with vaginal plaque were considered on the first day of pregnancy and were used to form experimental groups. For each group—young, middle‐aged, and old female mice—Days 5, 6, and 8 of pregnancy subgroups were created (Table 1). To determine the number of mice needed for the experiments, a power analysis was performed using the G*Power 3.1.9.4 program. An effect size of 0.9 (large effect) was used for the sample calculation. When the numbers in each experimental group were equal, it was determined that 10 mice were needed per group at an alpha level of 0.05 and a power of 0.95. Implantation sites obtained at Days 5, 6, and 8 of pregnancy were stored in liquid nitrogen and formalin for Western blotting and immunohistochemistry, respectively.

**TABLE 1:** Experimental animal groups.

Young female mice group (12‐week)	Middle‐aged female mice group (20‐week)	Old female mice group (26–32‐week)
5th day of pregnancy (*n* = 4)	5th day of pregnancy (*n* = 4)	5th day of pregnancy (*n* = 4)
6th day of pregnancy (*n* = 4)	6th day of pregnancy (*n* = 4)	6th day of pregnancy (*n* = 4)
8th day of pregnancy (*n* = 4)	8th day of pregnancy (*n* = 4)	8th day of pregnancy (*n* = 4)

### 2.2 Immunohistochemistry

Tissue sections were incubated with rabbit polyclonal anti‐pIncenp (Affinity Biosciences #AF8485) at 1:100 dilution, rabbit polyclonal anti‐Aurora Kinase B (Abcam #ab2254) at 1:100 dilution, rabbit polyclonal anti‐Survivin (Cell Signaling #2808S) at 1:50 dilution, rabbit polyclonal anti‐Borealin (MyBioSource #MBS127771) at 1:100 dilution, and rabbit polyclonal anti‐Ki‐67 (ProteinTech #28074‐1‐AP) at 1:1000 dilution, incubated at 4°C overnight. Subsequently, the samples were incubated with biotinylated anti‐rabbit secondary antibody (dilution: 1:500) (#BA‐1000, Vector Laboratories, Burlingame, USA) in a humidified environment at 25°C for 45 min. The samples were washed with PBS and incubated for 20 min with streptavidin peroxidase (# TS‐125‐HR; Thermo Scientific/Lab Vision). Sections were treated with DAB (Di Amino Benzidine) chromogen (#D4168, Sigma Aldrich, St. Louis, MO, USA) and then counterstained with Hematoxylin (#109249 Merck, Darmstadt, Germany). For each group, photographs were taken from the luminal epithelium, glandular epithelium, and stroma of six different mice. DAB, hematoxylin, and background images were obtained after “color deconvolution” on the ×400 photographs for each section. DAB concentrations in the sections were measured. Mathematical values of DAB intensities were compared statistically.

### 2.3 Sodium Dodecyl Sulfate (SDS) Polyacrylamide Gel Electrophoresis and Western Blotting

Protein extraction and immunoblot analysis were performed as previously described (Acar et al. 2012). Membranes were incubated overnight at +4°C with a 1:1000 dilution of rabbit polyclonal anti‐pIncenp (Affinity Biosciences #AF8485), a 1:500 dilution of rabbit polyclonal anti‐Aurora Kinase B (Abcam #ab2254), a 1:1000 dilution of rabbit polyclonal anti‐Survivin (Cell Signaling‐2808S), and a 1:500 dilution of rabbit polyclonal anti‐Borealin (MyBioSource MBS127771). pIncenp, Aurora Kinase B, and Borealin primary antibodies were diluted in 5% non‐fat powdered milk, while Survivin primary antibody was diluted in 5% BSA. HRP‐conjugated (horseradish peroxidase) (#PI‐1000, Vector Laboratories, Burlingame, USA) was used as a secondary antibody. It was diluted in 5% BSA for Survivin and in 5% non‐fat powdered milk for pIncenp, Aurora Kinase B, and Borealin. The membranes were also treated with Beta‐actin antibody (#4970, Cell Signaling Technology, Danvers, MA, USA) as an internal control. The bands were measured using NIH Image analysis software (ImageJ, Version 1.36b; National Institutes of Health, MD, USA). Then, the expression levels of pIncenp, Aurora Kinase B, Survivin, and Borealin were determined by normalizing their results to Beta‐actin. Consequently, the quantities of these proteins were assessed. The experiments were repeated three times.

### 2.4 Statistical Analysis

A one‐way analysis of variance (ANOVA) was performed to evaluate differences among groups. Post hoc comparisons were subsequently carried out using Tukey's Honestly Significant Difference (HSD) test. A *p*‐value < 0.05 was considered indicative of statistical significance. Data are reported as mean ± standard error of the mean (SEM).

## 3 Results

### 3.1 Implantation Site Numbers of Mice in the Experimental Groups

Table 2 shows the average number of implantation sites on Days 5, 6, and 8 of pregnancy in mice aged 12, 20, and 26–32 weeks.

**TABLE 2:** Mean and SEM values of the number of implantation sites obtained approximately on the 5th, 6th, and 8th days of pregnancy in 12, 20, and 26–32 week pregnant mice.

Days of pregnancy	12 weeks pregnant	20 weeks pregnant	26–32 weeks pregnant
D5	9.75 ± 0.25	8.25 ± 0.25	7.0 ± 0.57
D6	9 ± 0.40	8.25 ± 0.25	6.0 ± 0.50
D8	9.25 ± 9.47	6.75 ± 0.47	6.0 ± 0.50

**FIGURE 1:**
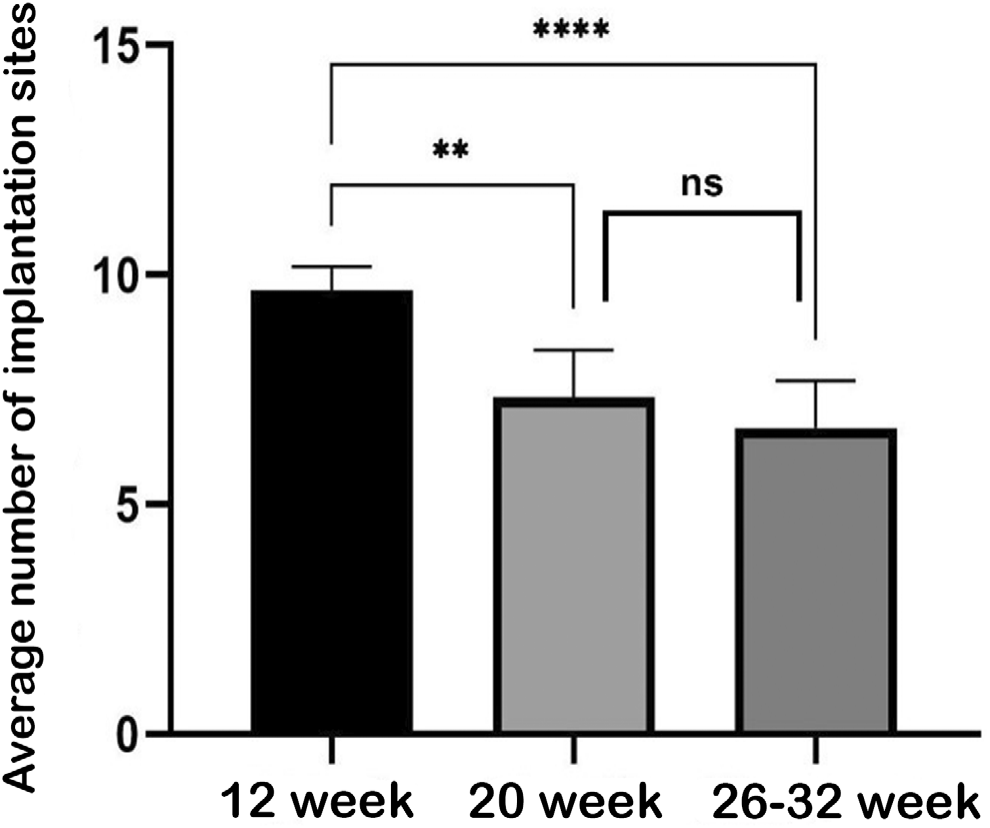
Analysis of the average number of implantation sites on Days 5, 6, and 8 of pregnancy in 12, 20, and 26–32 week old mice (****; *p* < 0.0001).

The number of all implantation sites on Days 5, 6, and 8 of pregnancy was compared among 12‐week‐old, 20‐week‐old, and 26–32‐week‐old mice. Compared to 12‐week‐old mice, a statistically significant decrease in the number of implantation sites was observed in both 20‐week‐old (*p* = 0.9918) and 26–32‐week‐old (*p* < 0.0001) mice. However, no statistically significant difference was found between 20‐week‐old and 26–32‐week‐old mice (*p* > 0.05) (Figure 1).

### 3.2 Immunohistochemistry Results

#### 3.2.1 pIncenp Immunohistochemistry Results

pINCENP localization and expression were evaluated and statistically analyzed in the luminal epithelium, glandular epithelium, and stroma of the implantation sites of the 12‐week, 20‐week, and 26–32‐week‐old experimental groups on the 5th, 6th, and 8th days of pregnancy. According to these results:

##### 3.2.1.1 On the 5th Day of Pregnancy

While no statistically significant difference was observed in pIncenp expression in the luminal epithelium between the 12‐week and 20‐week‐old experimental groups (*p* > 0.05), a statistically significant increase was observed in the 26–32‐week‐old mice compared to the 12‐week‐old and 20‐week‐old mouse groups (*p* < 0.0001) (Figure 1I).

There was a statistically significant increase in pIncenp expression in the glandular epithelium in the 20‐week (*p* = 0.0002) and 26–32‐week‐old (*p* = 0.0074) mice groups compared to the 12‐week‐old mice group. However, no statistically significant difference was observed in 26–32‐week‐old mice compared to 20‐week‐old mice (*p* > 0.05) (Figure 1I).

A statistically significant increase in pIncenp expression in the stroma was observed in the 20‐week (*p* < 0.0001) and 26–32‐week‐old (*p* < 0.0001) mice groups compared to the 12‐week‐old mice group. A statistically significant increase was also observed in 26–32‐week‐old mice compared to 20‐week‐old mice (*p* < 0.0001) (Figure 1I).

##### 3.2.1.2 On the 6th Day of Pregnancy

**FIGURE 2:**
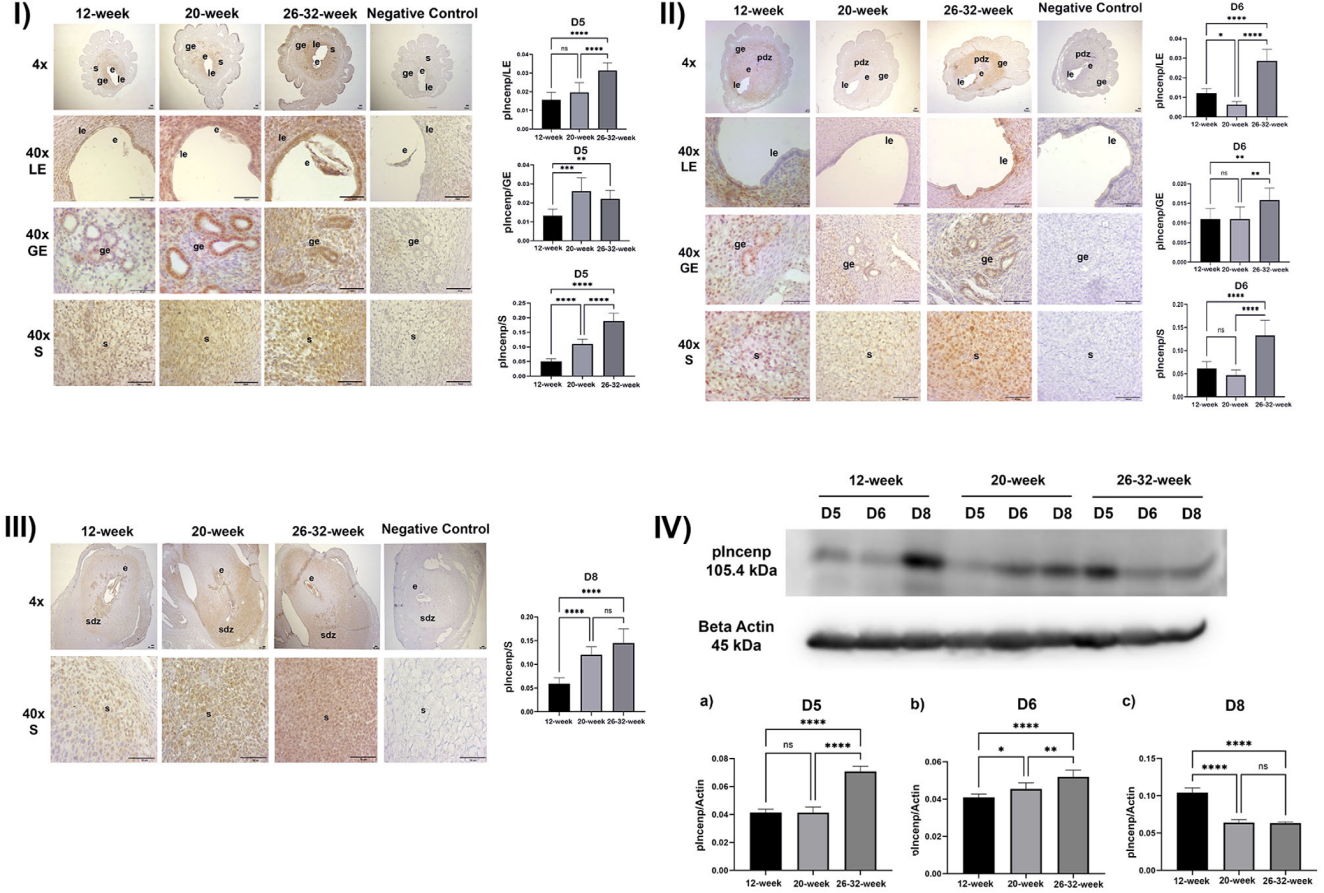
(I) pIncenp expression in the luminal epithelium, glandular epithelium, and stroma and ImageJ analysis at implantation sites on Day 5 of pregnancy in 12‐week‐old, 20‐week‐old and 26–32‐week‐old female mice (le, luminal epithelium; be, glandular epithelium; str, stroma; e, embryo; ****p < 0.0001, ns, not significant, Scala bar: 50 μm). (II) pIncenp expression in the luminal epithelium, glandular epithelium and stroma and ImageJ analysis at implantation sites on Day 6 of pregnancy in 12‐week‐old, 20‐week‐old and 26–32‐week‐old female mice (le, luminal epithelium; be, glandular epithelium; str, stroma; e, embryo; pdz, primary decidual zone, ****p < 0.0001, ns, not significant, Scala bar: 50 μm). (III) pIncenp expression in the luminal epithelium, glandular epithelium and stroma and ImageJ analysis at implantation sites on Day 8 of pregnancy in 12‐week‐old, 20‐week‐old and 26–32‐week‐old female mice (le, luminal epithelium; be, glandular epithelium; str, stroma; e, embryo; pdz, primary decidual zone; sdz, secondary decidual zone; ****p < 0.0001, ns, not significant, Scala bar: 50 μm). (IV) pIncenp/Actin Western blot analysis results at implantation sites on Days 5, 6, and 8 of pregnancy in 12‐week‐old, 20‐week‐old, and 26–32‐week‐old mice (****p < 0.0001, ns, not significant).

There was a statistically significant decrease in pIncenp expression in the luminal epithelium in 20‐week‐old mice compared to 12‐week‐old mice (*p* = 0.0132). A statistically significant increase in pIncenp expression was determined in the 26–32‐week‐old mice group compared to the 12 and 20‐week‐old groups (*p* < 0.0001) (Figure 2II).

While no statistically significant difference was observed in pIncenp expression in the glandular epithelium between 12‐week and 20‐week‐old experimental groups (*p* > 0.05), a statistically significant increase was observed in 26–32‐week‐old mice compared to 12‐week‐old (*p* = 0.0092) and 20‐week‐old (*p* = 0.0093) groups (Figure 2II).

No statistically significant difference was observed in pIncenp expression in the stroma between the 12‐week and 20‐week‐old experimental groups (*p* > 0.05). A statistically significant increase was observed in 26–32‐week‐old mice compared to 12‐week‐old and 20‐week‐old mice (*p* < 0.0001) (Figure 2II).

##### 3.2.1.3 On the 8th Day of Pregnancy

A statistically significant increase in pIncenp expression in the stroma was observed in the 20‐week and 26–32‐week groups compared to the 12‐week group (*p* < 0.0001). There was no statistically significant difference between the 20‐week and 26–32‐week groups (*p* > 0.05) (Figure 2III).

#### 3.2.2 Aurora B Immunohistochemistry Results

Aurora B localization and expression were evaluated and statistically analyzed in the luminal epithelium, glandular epithelium, and the stroma of the implantation sites of the 12‐, 20‐, and 26–32‐week experimental groups on the 5th, 6th, and 8th days of pregnancy. According to these results:

##### 3.2.2.1 On the 5th Day of Pregnancy

**FIGURE 3:**
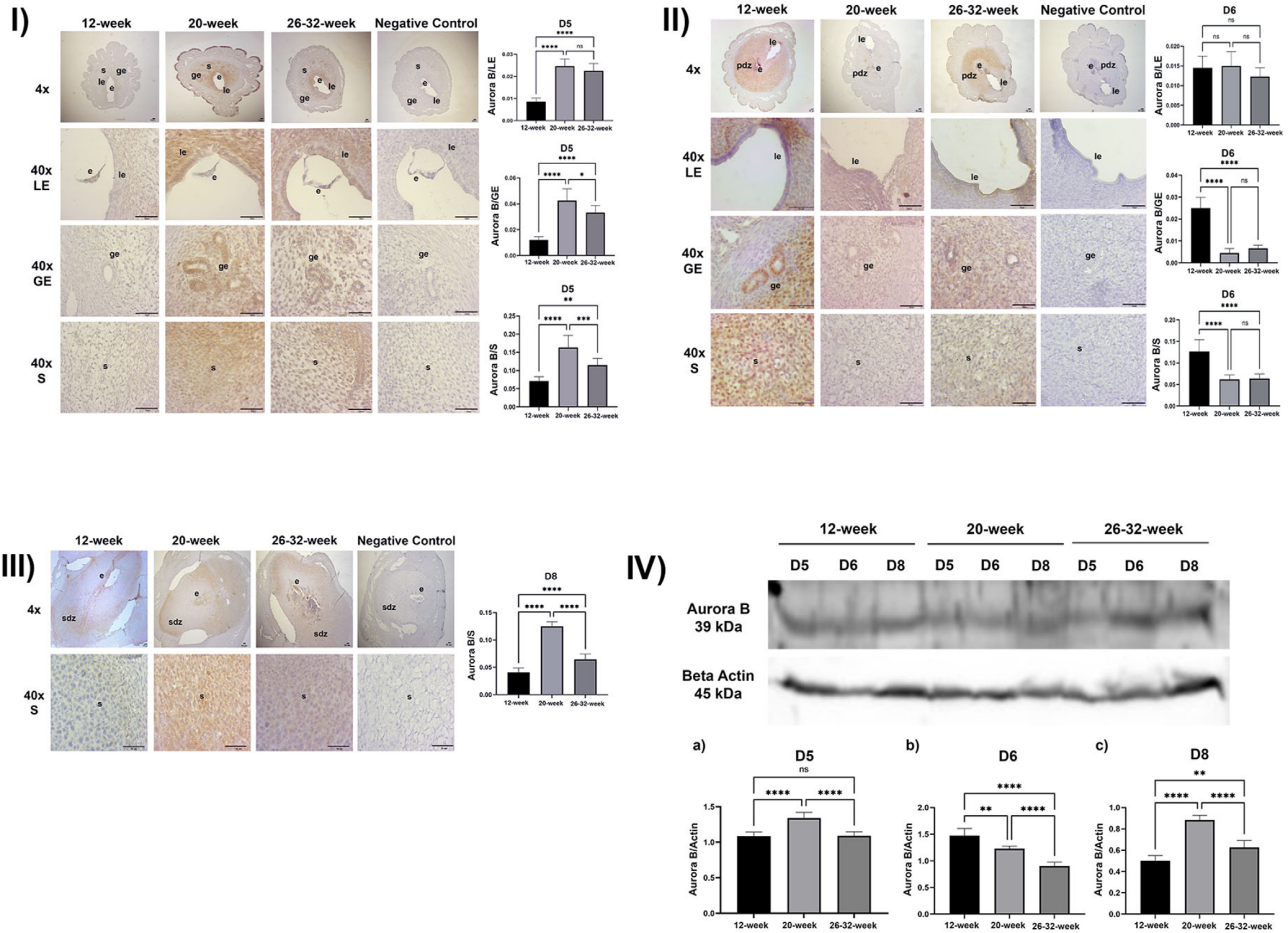
(I) Aurora B expression in the luminal epithelium, glandular epithelium, and stroma and ImageJ analysis at implantation sites on Day 5 of pregnancy in 12‐week‐old, 20‐week‐old and 26–32‐week‐old female mice (le, luminal epithelium; be, glandular epithelium; str, stroma; e, embryo; ****p < 0.0001, ns, not significant; Scala bar: 50 μm). (II) Aurora B expression in the luminal epithelium, glandular epithelium, and stroma and ImageJ analysis at implantation sites on day 6 of pregnancy in 12‐week‐old, 20‐week‐old and 26–32‐week‐old female mice (le, luminal epithelium; be, glandular epithelium; str, stroma; e, embryo; pdz, primary decidual zone, ****p < 0.0001, ns, not significant, Scala bar: 50 μm). (III) Aurora B expression in the luminal epithelium, glandular epithelium and stroma and ImageJ analysis at implantation sites on Day 8 of pregnancy in 12‐week‐old, 20‐week‐old and 26–32‐week‐old female mice (le, luminal epithelium; be, glandular epithelium; str, stroma; e, embryo; pdz, primary decidual zone; sdz, secondary decidual zone, ****p < 0.0001, ns, not significant, Scala bar: 50 μm). (IV) Aurora B/Actin Western blot analysis results at implantation sites on Days 5, 6, and 8 of pregnancy in 12‐week‐old, 20‐week‐old and 26–32‐week‐old mice (****p < 0.0001, ns, not significant).

A statistically significant increase in Aurora B expression in the luminal epithelium was observed in the 20 and 26–32‐week experimental groups compared to the 12‐week experimental group (*p* < 0.0001). There was no statistically significant difference between the 20‐week and 26–32‐week experimental groups (*p* > 0.05) (Figure 3I).

A statistically significant increase in Aurora B expression in the glandular epithelium was observed in the 20 and 26–32‐week experimental groups compared to the 12‐week experimental group (*p* < 0.0001). A statistically significant decrease was observed in the 26–32‐week experimental group compared to the 20‐week experimental group (*p* = 0.0188) (Figure 3I).

A statistically significant increase in Aurora B expression in the stroma was observed in the 20‐week (*p* < 0.0001) and 26–32‐week (*p* = 0.0026) experimental groups compared to the 12‐week experimental group. A statistically significant decrease was observed in the 20‐week experimental group compared to the 26–32‐week experimental group (*p* = 0.0009) (Figure 3I).

##### 3.2.2.2 On the 6th Day of Pregnancy

No statistically significant difference in Aurora B expression was observed between the experimental groups in the luminal epithelium (*p* > 0.05) (Figure 3II).

Aurora B expression in the glandular epithelium was statistically significantly decreased in the 20‐ and 26–32‐week experimental groups compared to the 12‐week experimental group (*p* < 0.0001). There was no statistically significant difference between the 20‐ and 26–32‐week experimental groups (*p* > 0.05) (Figure 3II).

Aurora B expression in the stroma was statistically significantly decreased in the 20‐ and 26–32‐week experimental groups compared to the 12‐week experimental group (*p* < 0.0001). There was no statistically significant difference between the 20‐ and 26–32‐week experimental groups (*p* > 0.05) (Figure 3II).

##### 3.2.2.3 On the 8th Day of Pregnancy

Aurora B expression in the stroma was statistically significantly increased in the 20‐ and 26–32‐week experimental groups compared to the 12‐week experimental group (*p* < 0.0001). A statistically significant decrease was observed in the 26–32‐week experimental group compared to the 20‐week experimental group (*p* < 0.0001) (Figure 3III).

#### 3.2.3 Survivin Immunohistochemistry Results

Survivin localization and expression were evaluated and statistically analyzed in the luminal epithelium, glandular epithelium, and the stroma of the implantation sites of the 12‐, 20‐, and 26–32‐week experimental groups on the 5th, 6th, and 8th days of pregnancy. According to these results:

##### 3.2.3.1 On the 5th Day of Pregnancy

**FIGURE 4:**
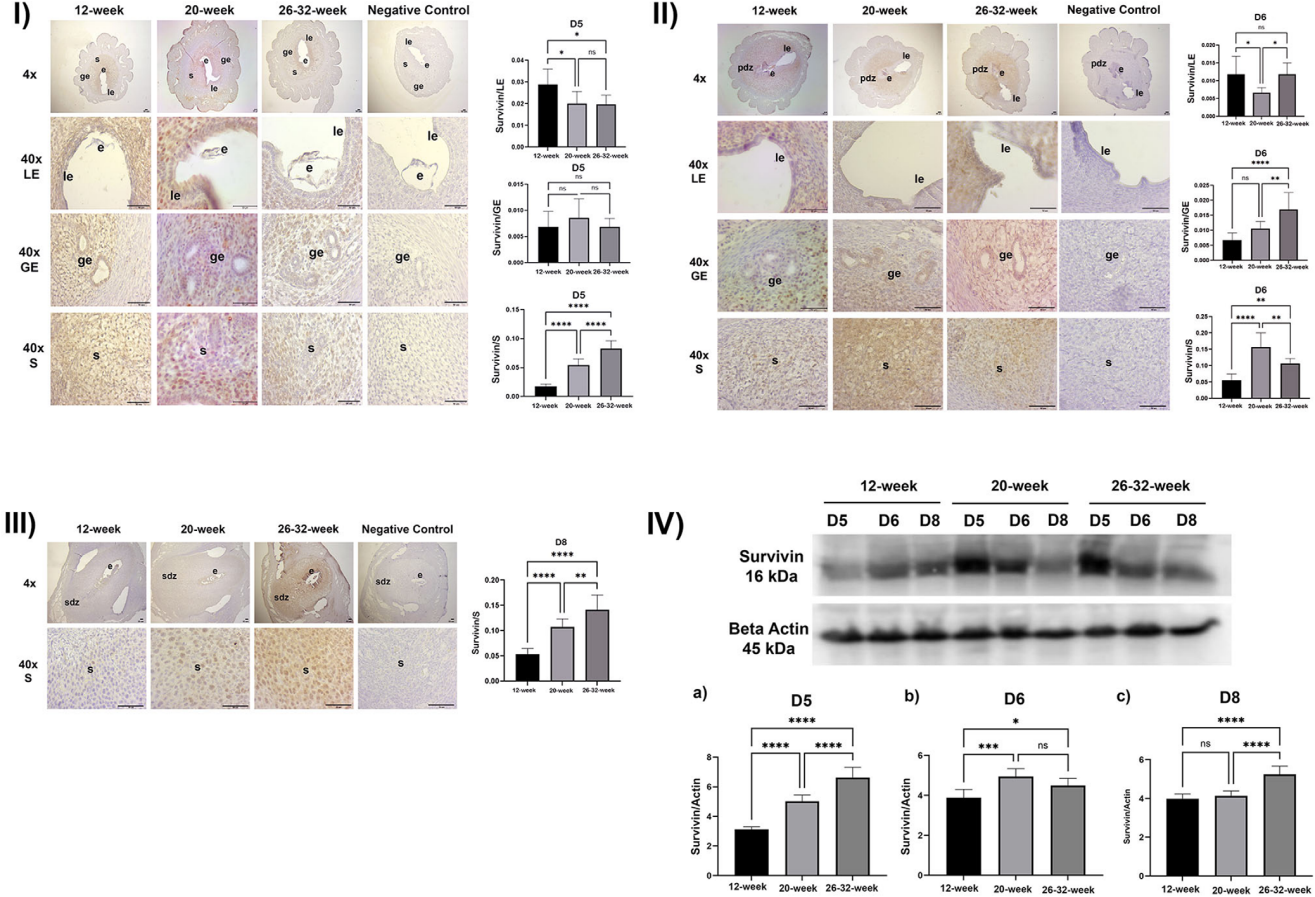
(I) Survivin expression in the luminal epithelium, glandular epithelium, and stroma and ImageJ analysis at implantation sites on Day 5 of pregnancy in 12‐week‐old, 20‐week‐old and 26–32‐week‐old female mice (le, luminal epithelium; be, glandular epithelium; str: stroma; e: embryo; ****p < 0.0001, ns, not significant; Scala bar: 50 μm). (II) Survivin expression in the luminal epithelium, glandular epithelium and stroma and ImageJ analysis at implantation sites on Day 6 of pregnancy in 12‐week‐old, 20‐week‐old and 26–32‐week‐old female mice (le, luminal epithelium; be, glandular epithelium; str, stroma; e, embryo; pdz, primary decidual zone, ****p < 0.0001, ns, not significant; Scala bar: 50 μm). (III) Survivin expression in the luminal epithelium, glandular epithelium and stroma and ImageJ analysis at implantation sites on day 8 of pregnancy in 12‐week‐old, 20‐week‐old, and 26–32‐week‐old female mice (le, luminal epithelium; be, glandular epithelium; str, stroma; e, embryo; pdz, primary decidual zone; sdz, secondary decidual zone, ****p < 0.0001, ns, not significant, Scala bar: 50 μm). (IV) Survivin/Actin Western blot analysis results at implantation sites on Days 5, 6, and 8 of pregnancy in 12‐week‐old, 20‐week‐old, and 26–32‐week‐old mice (****p < 0.0001, ns: not significant).

Survivin expression in the luminal epithelium was significantly decreased in the 20‐week (*p* = 0.0159) and 26–32‐week (*p* = 0.0111) experimental groups compared to the 12‐week experimental group. There was no statistically significant difference between the 20‐ and 26–32‐week experimental groups (*p* > 0.05) (Figure 4I).

No statistically significant difference was observed between the experimental groups for Survivin expression in the glandular epithelium (*p* > 0.05) (Figure 4I).

Survivin expression in the stroma was statistically significantly increased in the 20‐ and 26–32‐week experimental groups compared to the 12‐week experimental group (*p* < 0.0001). A statistically significant increase was observed in the 26–32‐week experimental group compared to the 20‐week experimental group (*p* < 0.0001) (Figure 4I).

##### 3.2.3.2 On the 6th Day of Pregnancy

Survivin expression in the luminal epithelium was statistically significantly decreased in the 20‐week experimental group compared to the 12‐week experimental group (*p* = 0.0188). No statistically significant difference was observed between the 12‐ and 26–32‐week experimental groups (*p* > 0.05). A statistically significant increase was observed in the 26–32‐week experimental group compared to the 20‐week experimental group (*p* < 0.0192) (Figure 4II).

There was no statistically significant difference in Survivin expression in the glandular epithelium between the 12‐ and 20‐week experimental groups (*p* > 0.05). A statistically significant increase was observed in the 26–32‐week experimental group compared to the 12‐week (*p* < 0.0001) and 20‐week (*p* = 0.0074) experimental groups (Figure 4II).

Survivin expression in the stroma was statistically significantly increased in the 20‐week (*p* < 0.0001) and 26–32‐week (*p* = 0047) experimental groups compared to the 12‐week experimental group. A statistically significant decrease was observed in the 26–32‐week experimental group compared to the 20‐week experimental group (*p* = 0.0052) (Figure 4II).

##### 3.2.3.3 On the 8th Day of Pregnancy

Survivin expression in the stroma was statistically significantly increased in the 20‐ and 26–32‐week experimental groups compared to the 12‐week experimental group (*p* < 0.0001). A statistically significant increase was observed in the 26–32‐week experimental group compared to the 20‐week experimental group (*p* < 0.0001) (Figure 4III).

#### 3.2.4 Borealin Immunohistochemistry Results

Borealin localization and expression were evaluated and statistically analyzed in the luminal epithelium, glandular epithelium, and the stroma of the implantation sites of the 12‐, 20‐, and 26–32‐week experimental groups on the 5th, 6th, and 8th days of pregnancy. According to these results:

##### 3.2.4.1 On the 5th Day of Pregnancy

**FIGURE 5:**
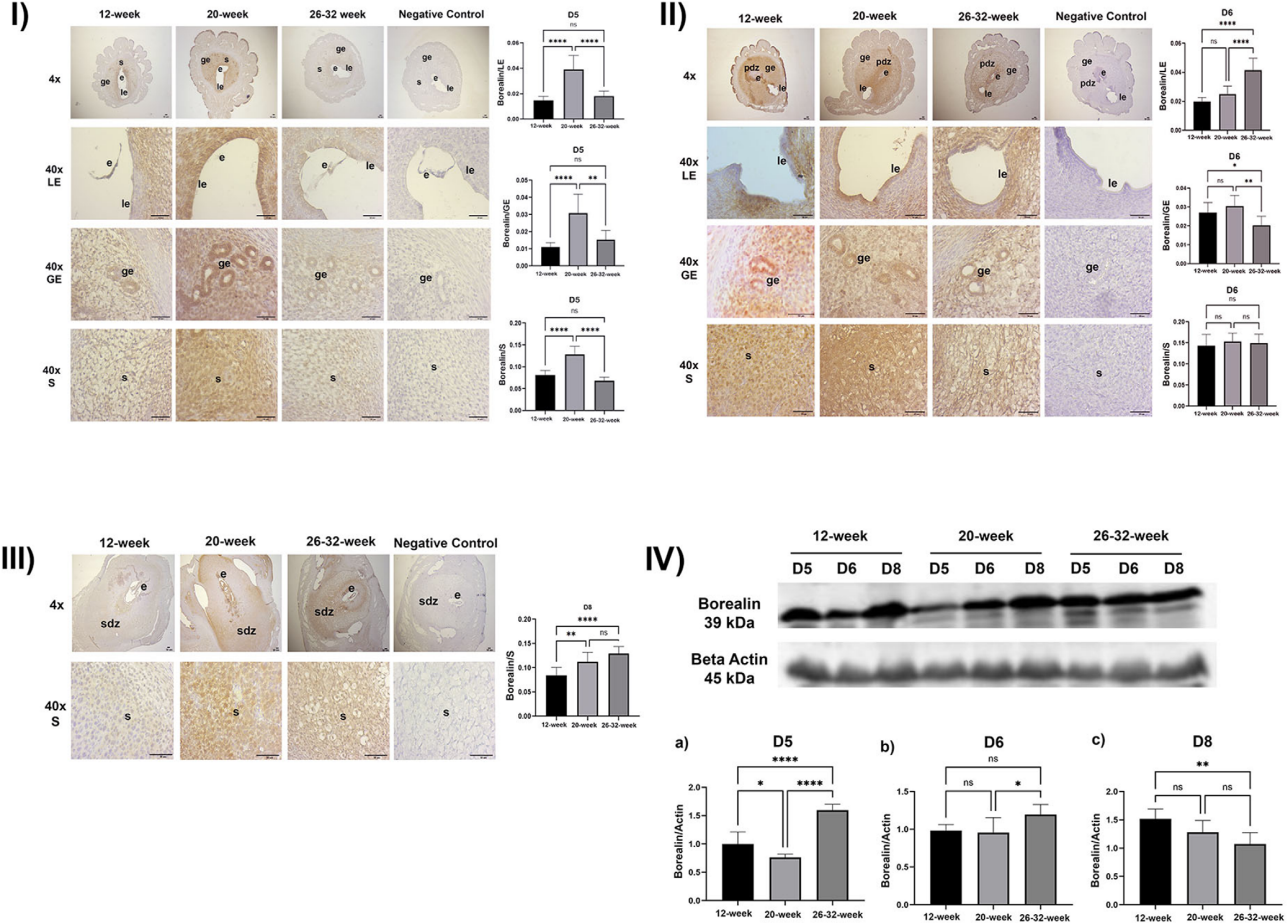
(I) Borealin expression in the luminal epithelium, glandular epithelium and stroma and ImageJ analysis at implantation sites on Day 5 of pregnancy in 12‐week‐old, 20‐week‐old and 26–32‐week‐old female mice (le, luminal epithelium; be, glandular epithelium; str, stroma; e, embryo; ****p < 0.0001, ns: Not significant, Scala bar: 50 μm). (II) Borealin expression in the luminal epithelium, glandular epithelium, and stroma and ImageJ analysis at implantation sites on day 6 of pregnancy in 12‐week‐old, 20‐week‐old, and 26–32‐week‐old female mice (le, luminal epithelium; be, glandular epithelium; str, stroma; e, embryo; pdz, primary decidual zone, ****p < 0.0001, ns, not significant, Scala bar: 50 μm). (III) Borealin expression in the luminal epithelium, glandular epithelium, and stroma and ImageJ analysis at implantation sites on Day 8 of pregnancy in 12‐week‐old, 20‐week‐old, and 26–32‐week‐old female mice (le, luminal epithelium; be, glandular epithelium; str, stroma; e, embryo; pdz, primary decidual zone; sdz, secondary decidual zone, ****p < 0.0001, ns, not significant, Scala bar: 50 μm). (IV) Borealin/Actin Western blot analysis results at implantation sites on Days 5, 6 and 8 of pregnancy in 12‐week‐old, 20‐week‐old and 26–32‐week‐old mice (****p < 0.0001, ns, not significant).

Borealin expression in the luminal epithelium was statistically significantly increased in the 20‐week experimental group compared to the 12‐week experimental group (*p* < 0.0001). No statistically significant difference was observed between the 12‐ and 26–32‐week experimental groups (*p* > 0.05). A statistically significant decrease was observed in the 26–32‐week experimental group compared to the 20‐week experimental group (*p* < 0.0001) (Figure 5I).

Borealin expression in the glandular epithelium was statistically significantly increased in the 20‐week experimental group compared to the 12‐week experimental group (*p* < 0.0001). No statistically significant difference was observed between the 12‐week and 26–32‐week experimental groups (*p* > 0.05). A statistically significant decrease was observed in the 26–32‐week experimental group compared to the 20‐week experimental group (*p* = 0.0011) (Figure 5I).

Borealin expression in the stroma was statistically significantly increased in the 20‐week experimental group compared to the 12‐week experimental group (*p* < 0.0001). No statistically significant difference was observed between the 12‐ and 26–32‐week experimental groups (*p* > 0.05). A statistically significant decrease was observed in the 26–32‐week experimental group compared to the 20‐week experimental group (*p* < 0.0001) (Figure 5I).

##### 3.2.4.2 On the 6th Day of Pregnancy

There was no statistically significant difference in Borealin expression in the luminal epithelium between the 12‐ and 20‐week experimental groups (*p* > 0.05). A statistically significant increase was observed in the 26–32‐week experimental group compared to the 12‐week experimental group (*p* < 0.0001) and the 20‐week experimental group (*p* < 0.0001) (Figure 5II).

There is no statistically significant difference in Borealin expression in the glandular epithelium between the 12‐ and 20‐week experimental groups (*p* > 0.05). There was a statistically significant decrease in the 26–32‐week experimental group compared to the 12‐week experimental group (*p* = 0.0490) and the 20‐week experimental group (*p* < 0.0028) (Figure 5II).

There was no statistically significant difference in the expression of Borealin in the stroma between the experimental groups (*p* > 0.05) (Figure 5II).

##### 3.2.4.3 On the 8th Day of Pregnancy

A statistically significant increase in Borealin expression in the stroma was observed in the 20‐week (*p* = 0.0084) and 26–32‐week (*p* < 0.0001) experimental groups compared to the 12‐week experimental group. No statistically significant difference was observed between 20‐ and 26–32‐week experimental groups (*p* > 0.05) (Figure 5III).

#### 3.2.5 Ki‐67 Immunohistochemistry Results

Ki‐67 localization and expression were evaluated and statistically analyzed in the luminal epithelium, glandular epithelium, and the stroma of the implantation sites of the 12‐week, 20‐week, and 26–32‐week experimental groups on the 5th, 6th, and 8th days of pregnancy. According to these results:

##### 3.2.5.1 On the 5th Day of Pregnancy

**FIGURE 6:**
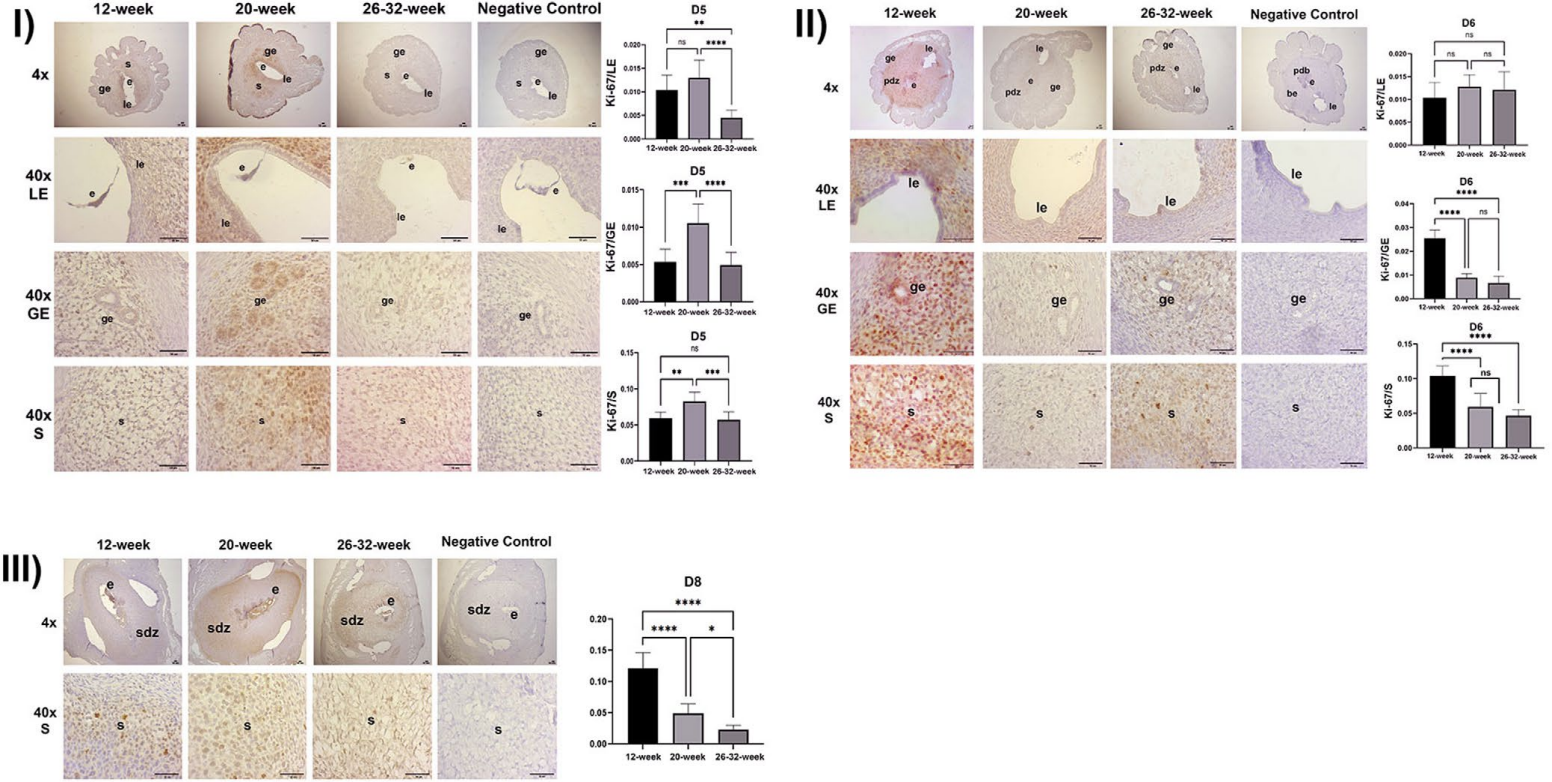
(I) Ki‐67 expression in the luminal epithelium, glandular epithelium, and stroma and ImageJ analysis at implantation sites on Day 5 of pregnancy in 12‐week‐old, 20‐week‐old and 26–32‐week‐old female mice (le, luminal epithelium; be, glandular epithelium; str, stroma; e, embryo, ****p < 0.0001, ns, not significant, Scala bar: 50 μm). (II) Ki‐67 expression in the luminal epithelium, glandular epithelium and stroma and ImageJ analysis at implantation sites on day 6 of pregnancy in 12‐week‐old, 20‐week‐old, and 26–32‐week‐old female mice (le, luminal epithelium; be, glandular epithelium; str, stroma; e, embryo, pdz: primary decidual zone, ****p < 0.0001, ns, not significant, Scala bar: 50 μm). (III) Ki‐67 expression in the luminal epithelium, glandular epithelium, and stroma and ImageJ analysis at implantation sites on Day 8 of pregnancy in 12‐week‐old, 20‐week‐old, and 26–32‐week‐old female mice (le, luminal epithelium; be, glandular epithelium; str, stroma; e, embryo, pdz, primary decidual zone; sdz, secondary decidual zone, ****p < 0.0001, ns, not significant, Scala bar: 50 μm).

There was no statistically significant difference in Ki‐67 expression in the luminal epithelium between the 12‐week and 20‐week experimental groups (*p* > 0.05). A statistically significant decrease was observed in the 26–32‐week experimental groups compared to the 12‐week (*p* = 0.0023) and 20‐week (*p* < 0.0001) experimental groups (Figure 6I).

A statistically significant increase in Ki‐67 expression in the glandular epithelium was found in the 20‐week experimental group compared to the 12‐week experimental group (*p* < 0.0001). No statistically significant difference was found between the 12‐week and 26–32‐week experimental groups (*p* > 0.05). A statistically significant decrease was observed in the 26–32‐week experimental group compared to the 20‐week experimental group (*p* < 0.0001) (Figure 6I).

Ki‐67 expression in the stroma was statistically significantly increased in the 20‐week experimental group compared to the 12‐week experimental group (*p* = 0.0011). No statistically significant difference was found between the 12‐ and 26–32‐week experimental groups (*p* > 0.05). A statistically significant decrease was observed in the 26–32‐week experimental group compared to the 20‐week experimental group (*p* = 0.0004) (Figure 6I).

##### 3.2.5.2 On the 6th Day of Pregnancy

No statistically significant difference in Ki‐67 expression was observed between the experimental groups in the luminal epithelium (*p* > 0.05) (Figure 6II).

A statistically significant decrease in Ki‐67 expression in the glandular epithelium was observed in the 20‐week and 26–32‐ experimental groups compared to the 12‐week experimental group (*p* < 0.0001). No statistically significant difference was found between the 20‐ and 26–32‐week experimental groups (*p* > 0.05) (Figure 6II).

A statistically significant decrease in Ki‐67 expression in the stroma was observed in the 20‐ and 26–32‐week experimental groups compared to the 12‐week experimental group (*p* < 0.0001). No statistically significant difference was found between the 20‐week and 26–32‐week experimental groups (*p* > 0.05) (Figure 6II).

##### 3.2.5.3 On the 8th Day of Pregnancy

A statistically significant decrease in Ki‐67 expression in the stroma was observed in the 20‐ and 26–32‐week experimental groups compared to the 12‐week experimental group (*p* < 0.0001). A statistically significant decrease was observed in the 26–32‐week experimental group compared to the 20‐week experimental group (*p* = 0.0224) (Figure 6III).

### 3.3 Western Blot Results

#### 3.3.1 pIncenp Western Blot Results

The amount of pIncenp protein on the 5th day of pregnancy was similar in the 12‐ and 20‐week groups, and no statistically significant difference was observed (*p* > 0.05). A statistically significant increase in pIncenp protein was observed in the 26–32‐week group compared to the 12‐week and 20‐week groups (*p* < 0.0001) (Figure 2IV).

On the 6th day of pregnancy, pIncenp protein showed a statistically significant increase in the 20‐week (*p* = 0.0486) and 26–32‐week groups (*p* < 0.0001) compared to the 12‐week group. A statistically significant increase in pIncenp protein was observed in the 26–32‐week group compared to the 20‐week group (*p* = 0.055) (Figure 2IV).

On the 8th day of pregnancy, a statistically significant decrease was observed in the 20‐week and 26–32‐week groups compared to the 12‐week group in terms of the pIncenp protein (*p* < 0.0001). No statistically significant difference was observed between the 20‐ and 26–32‐week groups (*p* > 0.05) (Figure 2IV).

#### 3.3.2 Aurora B Western Blot Results

The amount of Aurora B protein on the 5th day of pregnancy showed a statistically significant increase in the 20‐week group compared to the 12‐week group (*p* < 0.0001). No statistically significant difference was observed between the 12‐week group and the 26–32‐week group (*p* > 0.05). A statistically significant decrease was observed in the 26–32‐week group compared to the 20‐week group (*p* < 0.0001) (Figure 3IV).

On the 6th day of pregnancy, a statistically significant decrease was observed in Aurora B protein both in the 20‐week (*p* = 0.0010) and in the 26–32‐week groups (*p* < 0.0001) compared to the 12‐week group. A statistically significant decrease was observed in the 26–32‐week group compared to the 20‐week group (*p* < 0.0001) (Figure 3IV).

On the 8th day of pregnancy, the amount of Aurora B protein increased statistically significantly in the 20‐week (*p* < 0.0001) and 26–32‐week (*p* = 0.0027) groups compared to the 12‐week group. A statistically significant decrease was observed in the 26–32‐week group compared to the 20‐week group (*p* < 0.0001) (Figure 3IV).

#### 3.3.3 Survivin Western Blot Results

On the 5th day of pregnancy, the Survivin protein levels increased significantly in the 20‐week (*p* < 0.0001) and 26–32‐week (*p* < 0.0001) groups compared to the 12‐week group, and also in the 26–32‐week group compared to the 20‐week group (*p* < 0.0001) (Figure 4IV).

On the 6th day of pregnancy, Survivin protein increased in the 20‐week (*p* = 0.0008) and 26–32‐week (*p* = 0.0382) groups compared to the 12‐week group. Although Survivin protein decreased in the 26–32‐week group compared to the 20‐week group, no statistically significant difference was observed (*p* > 0.05) (Figure 4IV).

No statistically significant difference was observed between the 12‐week and 20‐week groups in Survivin protein amount on the 8th day of pregnancy (*p* > 0.05). A statistically significant increase was observed in the 26–32‐week group compared to the 12‐week (*p* < 0.0001) and 20‐week (*p* < 0.0001) groups (Figure 4IV).

#### 3.3.4 Borealin Western Blot Results

On the 5th day of pregnancy, the amount of Borealin protein decreased statistically significantly in the 20‐week group compared to the 12‐week group (*p* = 0.0295). A statistically significant increase was observed in the 26–32‐week group compared to the 12‐week group (*p* < 0.0001) and the 20‐week group (*p* < 0.0001) (Figure 5IV).

No statistically significant difference was observed between the 12‐week, 20‐week, and 12‐week, 26–32‐week groups for Borealin protein on the 6th day of pregnancy (*p* > 0.05). A statistically significant increase was observed in the 26–32‐week group compared to the 20‐week group (*p* = 0.0307) (Figure 5IV).

No statistically significant difference was observed between the 12‐week and 20‐week groups for Borealin protein on day 8 of pregnancy (*p* > 0.05). Compared to the 12‐week group, the amount of Borealin protein decreased statistically significantly in the 26–32‐week group (*p* = 0.0036). No statistically significant difference was observed between the 20‐ and 26–32‐week groups (*p* > 0.05) (Figure 5IV).

Overall, the Results show specific changes in uterine cell growth and chromosomal passenger complex (CPC) activity that vary with age and pregnancy stage during peri‐implantation days. On Day 5 of pregnancy, advancing maternal age was associated with decreased epithelial proliferative activity, as indicated by lower Ki‐67 expression, concomitant with increased stromal expression of pIncenp and Survivin, suggesting an early shift in cell‐cycle regulation at implantation sites. On Day 6, when decidualization is initiated, aging was characterized by sustained or increased expression of pIncenp, Borealin, and Survivin in the luminal epithelium and stroma, despite an overall decline in Ki‐67, suggesting an uncoupling between CPC activity and effective cell proliferation. By Day 8, during secondary decidual zone formation, Aurora B and Survivin expression were elevated in older mice, while Ki‐67 levels were markedly reduced, particularly in the stromal compartment. Collectively, these findings emphasize a progressive, age‐related imbalance between proliferative capacity and CPC regulation across pregnancy days, which may contribute to impaired implantation and decidualization in the aging uterus.

## 4 Discussion

Implantation of the embryo occurs when the uterus is receptive (Li et al. 2015). In early pregnancy, estrogen stimulates uterine epithelial cells for cell proliferation, while progesterone suppresses this estrogen‐induced cell proliferation (Chen et al. 2005). Reproductive capacity decreases with increasing age. Although it is thought that the main reason for the decrease in the pregnancy rate is ovarian aging, it is still debated whether uterine aging also contributes to this process. The implantation rate of blastocysts transferred from young donors to elderly pseudopregnant mice was lower (Boot and Muhlbock 1954; Talbert and Krohn 1966). Significant decreases were observed in both the pregnancy rate and the number of implantation sites in 12‐month‐old mice compared to 2‐month‐old mice (Li et al. 2017). A study was conducted to investigate the hypothesis that maternal age directly affects successful delivery. Three‐month‐old (young), 5‐month‐old (middle‐aged), and 8‐month‐old (elderly) female mice were used in the study. The birth timing and postnatal offspring sizes of these mice were examined. Their serum progesterone profiles, myometrium, and cervical functions were also examined. Elderly pregnant mice were found to have smaller offspring, and their gestation and delivery times were longer compared to 3‐month‐old mice. Cervical tissue from elderly pregnant mice was found to be more flexible and stretchable than that from young pregnant mice. No age‐related changes were observed in the enzymatic activities of mitochondrial electron transport chain complexes. This study demonstrated that 8‐month‐old mice could serve as a useful model for studying reproductive aging and contribute to our understanding of the relationship between aging and pregnancy success (Patel et al. 2017). In another study, stromal cells isolated from the proliferative phase of the menstrual cycle of women aged 25–35 and 36–46 were cultured. In the older age group, a decrease in stromal cell proliferation and in the expression of mRNA for the decidualization markers IGFBP1 and prolactin was observed. The researchers commented that aging affects the function of endometrial cells and their gene expression, as well as the rate of successful implantation (Berdiaki et al. 2022).

The chromosomal passenger complex members are proteins that move from the centromeres to the middle region of the spindle strands during mitosis. The CPC functions in the completion of cytokinesis at the end of mitosis by chromosome condensation and separation throughout mitosis (Adams et al. 2001). Some studies have confirmed that this complex has important roles in mitotic chromosome condensation, sister chromatid cohesion, spindle thread union, and cytokinesis completion. The CPC is present in a macromolecular complex, including the internal centromere protein (INCENP/Inner centromere protein), Aurora‐B kinase, Survivin (BIRC5), and Borealin (Zhang et al. 2009).

Some Kruppel‐like factor (Klf) family members responsible for cell proliferation in the mouse uterus are downregulated with aging. This results in the emergence of aging cells with decreased proliferation in uterine aging (Simmen et al. 2015). Our immunohistochemistry results showed that Ki‐67 decreased with aging in the luminal and glandular epithelium on the 5th day of pregnancy. Although no statistically significant difference was observed between the experimental groups in the luminal epithelium on the 6th day of pregnancy, a dramatic decrease in the glandular epithelium was noted at the 20‐week and 26–32‐week groups compared to the 12‐week group. On the 5th, 6th, and 8th days of pregnancy, it was observed that Ki‐67 expression decreased in the stroma of the implantation sites in parallel with aging. Our findings are consistent with information in the literature indicating that proliferation decreases with uterine aging.

The luminal epithelium, glandular epithelium, and stromal cells in the uterus respond differently to E_2_ and P_4_. E_2_ and P_4_ act together and regulate uterine cell proliferation and/or differentiation. In mice, uterine epithelial cells proliferate with the effect of pre‐implantation E_2_ on the 1st day of pregnancy. P_4_, which is released from the corpus luteum and whose levels are gradually increasing, initiates the proliferation of stroma cells from the 3rd day. Proliferation in the stroma is further stimulated by ovarian E_2_ secreted on the morning of the 4th day of pregnancy; proliferation of epithelial cells stops, and differentiation begins (Huet‐Hudson et al. 1989). With aging, estrogen levels decrease in women, especially during menopause (Davis and Baber 2022). In early pregnancy, estrogen stimulates uterine epithelial cells for proliferation, while progesterone suppresses this estrogen‐induced cell proliferation (Chen et al. 2005). It is known that progesterone and PR levels are also high with aging (Li et al. 2017).

On the 5th day of pregnancy, Ki‐67 expression in the stroma increased in the 20‐week group compared to the 12‐week young group and decreased in the 26–32‐week group compared to the 20‐week group. However, although the expression of the proliferation marker Ki‐67 decreased with aging, the expression of CPC members increased with aging. According to our Western blot results, while pIncenp, Borealin, and Survivin protein levels increased with aging, only Aurora B expression showed irregularity. Aurora B expression in the 20‐week group was higher than in the 12‐week group but lower than in the 26–32‐week group. This irregularity may be caused by steroid hormones that become irregular with aging and may affect the formation of the decidua, which begins to form on the 5th day of pregnancy. In light of all these data, we can interpret that the CPC is disrupted by aging and mitotic divisions, mutual interactions, and cellular differentiation processes occurring in the luminal, glandular epithelium, and stroma in the uterus, which affects embryo implantation and decidualization processes.

After the blastocyst binds to the uterine lumen epithelium, decidualization, defined as the differentiation of stromal cells into decidual cells, begins. This progesterone‐mediated cellular differentiation process is a prerequisite for a successful pregnancy (Ramathal et al. 2010). In rodents, the stromal cells that surround the blastocyst proliferate and differentiate to form the primary decidual zone (PDZ). PDZ begins to occur in the afternoon of the 5th day of pregnancy and is fully formed on the 6th day of pregnancy. PDZ disappears as the secondary decidual zone (SDZ) begins to form. SDZ is the layer of stroma cells that proliferate and differentiate around PDZ and is fully formed on the 8th day of pregnancy (Gellersen et al. 2007).

Several studies have reported that during the aging process in female mice, changes occur in ovarian hormone levels, which in turn lead to various alterations in the members of the chromosomal passenger complex. One study found that aging is associated with a decrease in the amount of estrogen receptors in the endometrial epithelium, stroma, and myometrium of female mice (Han et al. 1989). Another study observed that Muc1 and PR levels were high, and Hand2 levels were low in 12‐month‐old mice compared to 2‐month‐old mice on the 4th day of pregnancy (Li et al. 2017). With aging, the amount of Survivin in cells increases and accumulates, causing apoptosis resistance and negatively affecting the proliferation process (Al‐Khalaf and Aboussekhra 2013). Additionally, Survivin expression was observed to increase over time when estrogen was administered to ovariectomized mice. Conversely, Survivin expression decreased over time when progesterone was administered. These results suggest that Survivin expression is dynamically regulated by estrogen and progesterone in the uterus (Cho et al. 2020).

According to our immunohistochemistry and Western blot results, Survivin expression increased in the stroma of old mice compared to young mice on the 6th day of pregnancy. The literature indicates that Borealin and Survivin form a soluble complex in a 1:1 ratio. In the presence of INCENP N‐terminal peptide, this complex forms in a 1:1:1 ratio, Alan (Jeyaprakash et al. 2007). Moreover, on the 6th day of pregnancy, according to our immunohistochemistry and Western blot results in the stroma, we found that pIncenp and Borealin expressions increased in the elderly groups compared to the younger group, although they were not as sharp as Survivin. A study found that decidualization was impaired when the Aurora B kinase inhibitor Baracertib was administered in the artificial decidualization model in mice. In the in vitro decidualization model, it was observed that the cells were arrested at the G2/M phase when the inhibitor was applied (Wang et al. 2023). Although Survivin, pIncenp, and Borealin expressions increased with aging in the stromal sites on the 6th day of pregnancy, Aurora B expression decreased. Based on these results, decidualization might have been impaired in elderly mice on the 6th day of pregnancy because of aging and changes in the expression of chromosomal passenger complex members.

On the sixth day of pregnancy, the luminal epithelium exhibits no proliferative activity, since epithelial cells are eliminated through apoptosis after entosis (Li et al. 2015). Ki‐67 immunohistochemistry results in the luminal epithelium at Day 6 of pregnancy revealed no statistically significant difference among the young, middle‐aged, and elderly groups; nonetheless, an apparent increase in Ki‐67 expression was observed in 20‐ and 26–32‐week‐old mice relative to the 12‐week‐old group. However, our immunohistochemical analyses demonstrated a statistically significant upregulation of pIncenp, Borealin, and Survivin in the luminal epithelium of aging groups relative to younger groups on Day 6 of pregnancy. While Aurora B expression showed a non‐significant decrease in the aging group compared to the younger group, the observed alterations in CPC members' expression and the increased Ki‐67 levels in the luminal epithelium suggest that proliferative activity may persist in elderly mice on Day 6 of pregnancy.

On the 8th day of pregnancy, the primary decidual region disappears, and only the secondary decidual region remains. As pregnancy progresses, the secondary decidual region turns into a thin layer of cells called decidua capsularis. The mesometrial decidual cells then join to form the maternal part of the placenta, known as the decidua basalis. Defects that occur during the formation of the placenta can lead to health complications during pregnancy, such as preterm birth or pre‐eclampsia (Tsai et al. 2021). In a study, AurkB was observed to be more expressed in decidual cells between Days 6 and 7 of pregnancy. However, it was observed that the AurkB mRNA signal reduced in the decidual region on the 8th day of pregnancy (Wang et al. 2023). According to our immunohistochemistry results, Aurora B expression in the secondary decidual region was statistically significantly lower in the 12‐week group than in the 20‐ and 26–32‐week groups. Our Western blot results are also consistent with our immunohistochemistry results, as Aurora B expression in the young group on the 8th day of pregnancy is significantly lower than in the aging groups. In line with these data, Aurora B expression, which increases with aging, may be related to impaired decidualization. It is known that Survivin accumulates in cells with aging (Al‐Khalaf and Aboussekhra 2013). Our immunohistochemistry results on the 8th day of pregnancy showed that Survivin expression increased in the secondary decidual region with aging. Our Western blot results are also consistent with these results, and the expression of Survivin increases with aging. A study determined that an increase in nuclear Borealin expression was associated with a poor prognosis in gastric cancers. This increase was also correlated with an increase in Survivin expression (Chang et al. 2006). According to our immunohistochemistry results on the 8th day of pregnancy, the expression of nuclear Borealin increased in the aging groups compared to the young group, and Survivin expression was correlated with this increase. In line with the literature and our findings, aging and changes in the expression of members of the chromosomal passenger complex may lead to errors in the formation of the secondary decidual region.

Our study is the first to reveal the possible change of the chromosomal passenger complex due to aging at implantation and decidualization sites in mice in vivo. Although the age‐related changes in the expression and localization of chromosomal passenger complex (CPC) components were thoroughly assessed by immunohistochemistry and Western blotting, the study primarily provides descriptive observations. It lacks functional experiments that would directly show how CPC dysregulation causes implantation failure or hampers decidualization. Secondly, the analysis focused only on protein expression levels and their spatial localization. It did not include an evaluation of related transcriptional changes, post‐translational modifications, or kinase activity—especially Aurora B—which could provide further mechanistic insight. Third, uterine hormonal status was not directly measured, and circulating or local levels of estrogen and progesterone were not correlated with CPC expression patterns, which may be especially relevant given age‐related endocrine alterations. Additionally, although the age groups used are widely accepted in murine reproductive aging models, they may not fully capture the complexity and variability of uterine aging across the reproductive lifespan. Lastly, because this research was conducted solely in a mouse model, caution is warranted when extending the results to human implantation and reproductive aging. Future research on functional modulation of CPC components, hormone profiling, and analysis of human endometrial samples will be essential to clarify the role of CPC dysregulation in reproductive failure associated with aging. We think that these findings may be important for other studies to elucidate mechanisms such as implantation failure and recurrent pregnancy losses encountered in the clinic in the future.

## 5 Conclusion

Besides the healthy development of the embryo, several factors are pivotal for a successful pregnancy, including synchronous receptivity of the uterus, embryo‐uterus crosstalk, stromal cell differentiation into decidual cells, angiogenesis, and placental development. Although oocyte quality is thought to be the primary factor in fertility, uterine aging has also been shown to play a role in reproductive failure. In this study, the localization and expression of the chromosomal passenger complex (CPC) members pIncenp, Borealin, Survivin, and Aurora B at the implantation/desidualization sites of mice on Days 5, 6, and 8 of pregnancy were investigated by immunohistochemistry and Western blotting, and the expression of the proliferation marker Ki‐67 was determined by immunohistochemistry. These localization and expression changes were determined during the aging process. The CPC members showed dynamic changes in the uterus and implantation sites on different days of pregnancy and age. As a result, it is possible to say that the CPC may have roles during early pregnancy. Understanding the expression changes of members of the CPC with aging may lead to improved pregnancy success in women suffering from recurrent pregnancy loss or spontaneous abortion of unknown cause.

## Author Contributions

**Ezgi Golal:** conceptualization, investigation, formal analysis, writing original draft, visualization. **Cemre Nur Cemre Nur Balci:** methodology development, formal analysis. **Nuray Acar:** conceptualization, writing review and editing.

## Funding

This study was supported by The Scientific and Technological Research Council of Türkiye (TUBITAK), Ankara, Turkey. Project number: 222S872.

## Ethics Statement

All applicable international, national, and/or institutional guidelines for the care and use of animals were followed. The experimental protocol was approved by the Animal Care and Use Committee of Akdeniz University Faculty of Medicine (approval number: I568/2023.03.003).

## Consent

The authors have nothing to report.

## Conflicts of Interest

The authors declare no conflicts of interest.

## Data Availability

Available upon request. For requests, you can contact the corresponding author, Prof. Dr. Nuray ACAR.
